# Single-cell Transcriptomic Profiling Reveals Novel Stem Cell Markers for Corneal Limbal Epithelium Homeostasis

**DOI:** 10.21203/rs.3.rs-10594726/v1

**Published:** 2026-08-31

**Authors:** Qiang Zhou, Victor Guaiquil, Maria Paula Zappia, Jin-Hong Chang, Gongyu Tang, Mauricio Gonzalez Oyarzun, Minsu Cho, Joy Sarkar, Xiaowei Wang, Maxim Frolov, Elizaveta Benevolenskaya, Mark Rosenblatt

**Affiliations:** University of Illinois at Chicago; University of Illinois at Chicago; University of Illinois at Chicago; University of Illinois at Chicago; University of Illinois at Chicago; University of Illinois at Chicago; University of Illinois at Chicago; University of Illinois at Chicago; University of Illinois at Chicago; University of Illinois at Chicago; University of Illinois at Chicago; University of Illinois at Chicago

**Keywords:** Sox9, cornea, limbal stem cells, homeostasis, single-cell transcriptome analysis, marker

## Abstract

Sox9 is a well-established marker of skin epithelial hair follicle stem cells and has been observed in the corneal limbus of Sox9-EGFP transgenic mice. Using Sox9-EGFP as a reference marker, this study characterized the transcriptomic atlas of corneal limbal epithelial cells during homeostasis and identified novel markers of limbal stem cells (LSCs) by single-cell RNA sequencing (scRNA-seq). A total of 21,066 viable cells were profiled, revealing 13 distinct cell clusters with unique molecular signatures. The cluster enriched with EGFP-positive cells was identified as the putative LSCs population during corneal epithelial homeostasis. Comparative analysis of corneal LSCs and early differentiated cells revealed several differentially expressed genes (DEGs) strongly associated with stemness-related pathways, including Gabrp, Fmo1, Fmo2, and Alcam, which were identified as potential novel LSC markers. These genes were exclusively expressed in the limbus, as confirmed by quantitative PCR (qPCR), Western blotting, and immunofluorescence staining. This study delineated the heterogeneous cellular composition of the limbal epithelium and uncovered several novel candidate markers that may be critical for maintaining corneal epithelial homeostasis.

## Introduction

The cornea is a transparent, avascular tissue that is essential for refraction of light and for maintaining optimal visual acuity. Its surface is covered by a stratified epithelium that serves as a primary barrier, protecting the underlying stroma from environmental and mechanical insults. It is well established that the corneal epithelium undergoes complete renewal approximately every 2 weeks [1], a process driven by limbal stem cells (LSCs) located at the corneoscleral junction-limbus [2]. The preservation of corneal epithelial homeostasis depends on the functional integrity of these LSCs [3]. Disruption of corneal epithelial homeostasis can result in diminished visual acuity, ocular surface inflammation, dry eye disease, corneal neovascularization, and blindness [4].

The palisades of Vogt [5] and the epithelial crypt [6] are distinctive structures of the corneoscleral limbus, which are believed to function as an LSC niche. Within these niches, several genes, such as Sox9, Tspan7, Sox17, Runx1, Smad3, Atf3, Abcb5, H2ax, Pbk, and Plk3, are highly expressed and may be associated with the regulation of LSCs’ function [2]. Other putative LSC markers have also been reported, including ΔNp63α, Krt5, Krt19, Integrin β1, Integrin β4, Frizzled7, and ABCG2 [7]. Recently, several genes and transcription factors have been identified as novel putative LCS markers, including Gpha2, Ifitm3, Tspan7 [8], CD63, Bmi1, and TCF4 [9]. However, their specificity remains controversial, and there is still no universally accepted molecular marker for LSCs [10–14].

Transgenic mouse models have proven valuable for the lineage tracing of cells expressing genes associated with LSCs. Altshuler et al. used Krt15-confetti mice to track corneal epithelial cells expressing Krt15-EGFP and identified distinct LSC populations [15]. However, Krt15 is broadly expressed in both basal corneal and conjunctival epithelial cells [16] and is regarded as a marker of laterally differentiating epidermal keratinocytes [17], which limits its specificity as a definitive LSC marker for future research.

Sox9 is a critical gene involved in maintaining stem cell self-renewal across various tissues and plays a protective role against tissue damage [18–20]. Widely recognized as a marker of epidermal stem cells, Sox9 is indispensable for early stem cell specification, tissue development, and adult tissue homeostasis, and is essential for efficient epidermal wound healing. It is predominantly expressed in early slow-cycling or quiescent cells and contributes significantly to the development of all skin epithelial lineages [18, 21]. Studies have also demonstrated that Sox9 plays a critical role in maintaining LSCs’ quiescence and activation [22], which are essential for regulating the asymmetric cell-fate decisions required to maintain corneal epithelial homeostasis [23]. Our previous study also observed elevated Sox9 expression in corneal epithelial slow-cycling cells by the next-generation RNA sequencing and immunohistochemical staining in an inducible transgenic pulse-chase mouse [24].

In epithelial stem cell research, Sox9-EGFP transgenic mice have been widely utilized to investigate various epithelial stem cell populations. Studies in the small intestine and colon have demonstrated that Sox9-EGFP-positive cells possess multipotent potential within the intestinal epithelium [25]. Additionally, Sox9-EGFP expression is high in stem cells but diminished in transit-amplifying (TA) cells [26]. Furthermore, the Sox9-EGFP model has also been employed to track skin epithelial cell development and wound healing by monitoring EGFP fluorescence in hair follicles [27]. In our previous studies, we observed robust EGFP expression in the corneal limbus of the Sox9-EGFP transgenic mice. Although EGFP-positive cells are relatively rare, the high Sox9-EGFP expression in the mouse cornea makes this animal model a promising tool for investigating corneal limbal stem cells.

Single-cell RNA sequencing (scRNA-seq) has emerged as a powerful technology for investigating rare cell populations. This approach enables transcriptome profiling at the individual cell level while preserving information of the cells’ original identity [28]. It is particularly useful for analyzing tissues containing limited numbers of target cells and heterogeneous cell populations. By profiling individual cells, a comprehensive cellular atlas can be constructed, allowing comparison of gene expression profiles among distinct cell populations [29]. This innovative technique has enabled detailed investigation of LSCs during corneal epithelial cell homeostasis [15, 30, 31].

In this study, we performed single-cell transcriptomic profiling of corneal limbal epithelial cells obtained from Sox9-EGFP transgenic mice under homeostatic conditions. We characterized the gene expression profiles of Sox9-EGFP-positive cells, investigated transcriptional heterogeneity across cell populations, and focused on the genes associated with stem cell maintenance. Using gene enrichment and differential gene expression analyses, we identified differentially expressed genes (DEGs) by comparing the Sox9-EGFP cell cluster with other differentiated epithelial cell clusters, and validated their potential as novel LSCs markers during corneal epithelial homeostasis.

## Materials and Methods

### Animals

Sox9-EGFP transgenic mice (Male, 10–12 weeks old) were kindly provided by Dr. Elaine Fuchs’ laboratory at Rockefeller University. Male and female CD-1 mice (10–12 weeks old) were obtained from Jackson Laboratories. All animals were housed and bred in the Biologic Resources Laboratory at the University of Illinois Chicago (UIC) in accordance with National Institutes of Health (NIH) guidelines. All corneal tissues used in our experiments were collected after a combination of CO_2_ inhalation and cervical dislocation euthanized the mice. Experimental protocols were approved by the Institutional Animal Care and Use Committee (IACUC) of UIC and conducted in compliance with the NIH Guide for the Care and Use of Laboratory Animals, the Association for Research in Vision and Ophthalmology (ARVO) Statement for the Use of Animals in Ophthalmic and Vision Research, and the ARRIVE guidelines.

### Corneal epithelial cells isolation

Corneal limbal and central epithelial cells were isolated separately from the mice. After the corneal tissues were collected, the limbus at the peripheral cornea and the central cornea were separated by using a 2.0 mm trephine (Integra, Elmhurst, IL, USA). Limbal and central corneal epithelial cells were isolated according to our previously published protocols with adjustments [24, 32]. Briefly, corneal samples were incubated with 12.6 units of Dispase II (Roche Diagnostics, Basel, Switzerland) and 100 mM D-sorbitol (Sigma Aldrich, St. Louis, MO, USA) in CnT-50 medium (CELLnTEC, Bern, Switzerland) at 4°C. Detached epithelial sheets were collected with gentle mechanical assistance using a spatula to separate the corneal epithelium from the underlying stroma. Then, the cell sheets were dissociated into single cells by incubation with 0.05% trypsin and 0.03% EDTA (Life Technologies, NY, USA) at 37°C for 25 minutes. Enzymatic digestion was halted by adding CnT-50 medium supplemented with fetal calf serum (FCS) (Sigma-Aldrich, St. Louis, MO). The resulting cell suspension was filtered through a 35 μm cell strainer (Corning^™^ Falcon^™^ Round-Bottom Polystyrene Test Tubes). After two centrifugation and washing steps using PBS, single-cell suspensions were resuspended in 50 μL of DMEM supplemented with 10% FCS.

### Single-cell samples quality control, library generation, and RNA-sequencing

Because very few Sox9-EGFP-positive cells were present in the corneal limbus of Sox9-EGFP transgenic mice, we pooled corneal limbal epithelial cells from multiple mice to generate single samples for scRNA-seq. Specifically, two distinct samples were prepared, each consisting of cells isolated and pooled from both eyes of five Sox9-EGFP mice, with each sample representing 10 eyes. The quality of the isolated limbal epithelial cells was assessed by staining the samples with the cell viability dye, trypan blue, and acridine orange (AO)/propidium iodide (PI), using the LUNA-Fx7 automated cell counter (Logos Biosystems). Single-cell suspensions were diluted to 1,200 cells/μL and loaded into the Chromium X (10x Genomics) to target 10,000 cells per channel. For Gel Bead-in-emulsion (GEM) generation and barcoding, the samples were prepared following the 3’ gene expression Next GEM assay v3.1 with dual indexing (10xGenomics) in the Department of Biochemistry and Molecular Genetics at the University of Illinois Chicago College of Medicine. The quality of the amplified cDNA samples was evaluated using the High Sensitivity D5000 ScreenTape assay in the 4150 TapeStation system (Agilent). Libraries were constructed, and sequencing was performed on an Illumina NovaSeq 6000 (SP flow cell) and NovaSeq X Plus (10B) platform at the Roy J. Carver Biotechnology Center, University of Illinois at Urbana-Champaign.

### scRNA-sequencing data collection and processing

The scRNA-seq data were processed and mapped using Cell Ranger (version 7.0.1), a computational pipeline developed by 10x Genomics that performs read alignment, barcode processing, and gene expression quantification. For this, the raw sequencing data in FASTQ format were first demultiplexed and converted from BCL files using the mkfastq module, and index hopping was performed using the index-hop filter. Reads were subsequently aligned to the mouse reference genome (GRCm38) with gene annotations from the Ensembl database (version 102) via the align module, which employs the STAR aligner (version 2.7.10b) to generate BAM files containing mapped reads. The EGFP_bGhpolyA sequence (Supplementary Text) [33] was included in the reference genome to align the EGFP reads from Sox9-EGFP. The Cell Ranger count module was then used to produce a feature-barcode matrix quantifying gene expression for each cell. This module identifies cell barcodes and unique molecular identifiers (UMIs) from the mapped BAM file, producing a filtered BAM file comprising only cell-associated reads. The filtered BAM file serves to generate a digital expression matrix (DEM) that enumerates UMIs per gene in each cell. Quality control and normalization of the aggregated data were conducted using Cell Ranger’s aggr module, which integrates DEMs from multiple scRNA-seq libraries to generate a normalized expression matrix for downstream analysis.

### Bioinformatic Analysis of scRNA-seq Data and Differentially Expressed Genes (DEGs) Identification

The scRNA-seq data underwent quality control, normalization, feature selection, and clustering prior to downstream analyses. The Seurat v4.0 package and associated tools were used for bioinformatics processing, and batch effects across samples were corrected using the Harmony package. Cells exhibiting high mitochondrial gene content, low unique molecular identifier (UMI) counts, or low gene expression were excluded. Gene expression levels were normalized to the total UMI count per cell, then log-transformed using the “LogNormalize” function. Principal component analysis (PCA) was performed to capture major sources of variation in the dataset. Subsequently, cell subpopulations were identified using the “FindNeighbors” and “FindClusters” functions. t-SNE was used to visualize and compare cellular clusters across sample groups. Expression of marker genes within each cluster was visualized using dot plots. Cell cycle phase scoring was performed using canonical marker genes via the “CellCycleScoring” function. DEGs between clusters or between experimental conditions were identified using the “FindAllMarkers” or “FindMarkers” functions, respectively, with thresholds of adjusted p-value < 0.05, and absolute log_2_ fold change > 0.25, using the Wilcox test, and area under the curve (AUC) > 0.7.

### Gene Annotation and Functional Enrichment Analysis

DEG datasets were subjected to gene enrichment analysis using Metascape with default settings and clusterProfiler 4.0 [23, 34]. The analysis included GO/KEGG terms, canonical pathways, and hallmark gene sets, etc. The cumulative hypergeometric p-values and enrichment factors were calculated and used to filter. Remaining significant terms were then hierarchically clustered into a tree based on Kappa-statistical similarities among their gene memberships, with a Kappa score threshold of 0.3 used to define term clusters.

### Total RNA Extraction, Reverse Transcription, and Quantitative Real-Time PCR (RT-qPCR)

Corneal limbal and central corneal epithelial cells were isolated separately from CD-1 wild-type mice as described above to validate the expression of candidate LSCs markers. Total RNA was extracted using the RNeasy Micro Plus Kit (Qiagen, Hilden, Germany), quantified with a NanoDrop ND-1000 spectrophotometer, and stored at −80°C. First-strand cDNA synthesis was performed using the Applied Biosystems Reverse Transcription Kit (catalog number 4368814). Quantitative real-time PCR was performed on the Applied Biosystems QuantStudio 7 Flex Real-Time PCR System using SYBR Green reagents. Primer pairs were designed with the IDT PrimerQuest Tool (IDT, Coralville, IA, USA) and listed in Supplementary Table S1. Relative gene expression levels were analyzed using the comparative threshold cycle (ΔΔCt) method and normalized to the housekeeping gene GAPDH.

### Western Blot Analysis

Corneal limbal and central corneal epithelial cells were harvested respectively from CD-1 wild-type mice as described above. Total proteins were immediately extracted from the isolated epithelial cells. Tissues were homogenized and lysed in modified RIPA buffer (20 mM Tris-HCl, pH 7.4; 150 mM NaCl; 1 mM EDTA; 1 mM EGTA; 1% IGEPAL; 2.5 mM sodium pyrophosphate; 1 mM β-glycerophosphate), supplemented with protease and phosphatase inhibitor cocktails (Sigma-Aldrich, St. Louis, MO). Lysates were centrifuged at 10,000 × g for 15 minutes at 4°C, and the supernatants were collected. Protein concentrations were determined using the BioRad DC Protein Assay. For Western blotting, 50 μg protein per sample was resolved on 4–12% Tris-glycine SDS-PAGE gels and transferred to 0.2 μm nitrocellulose membranes. Membranes were blocked with 5% milk for 1 hour, followed by overnight incubation at 4°C with primary antibodies against GAPDH (Cell Signaling Technology, 14C10, 1:1000), Krt-8 (Proteintech, 10384–1-AP, 1:2000), Krt-18 (Proteintech, 10830–1-AP, 1:1000), Anxa-3 (Proteintech, 17957–1-AP, 1:1000), Fmo-1 (Novus Biologicals, NBP2–16511, 1:500), Fmo-2 (Proteintech, 15725–1-AP, 1:500), Gabrp (Proteintech, 17957–1-AP, 1:500), and Alcam (Proteintech, 21972–1-AP, 1:500). After washing, membranes were incubated with IRDye 800CW-conjugated goat anti-rabbit secondary antibodies (1:5,000) for 1 hour at room temperature. Blots were imaged using a Li-Cor Odyssey Infrared Imager, and band intensities were quantified with Fiji (NIH ImageJ) and normalized to GAPDH after background subtraction.

### Immunofluorescence Staining and Antibodies

Corneas of CD-1 wild-type mice were embedded in optimal cutting temperature (OCT) compound, sectioned, and fixed in 4% paraformaldehyde at room temperature for 10 minutes. Sections were permeabilized with 0.5% Triton X-100 in phosphate-buffered saline (PBS) for 10 minutes and blocked with 10% goat serum for 1 hour. The following primary antibodies were applied overnight at 4°C, including: Krt-8 (Proteintech, 10384–1-AP, 1:200), Krt-18 (Proteintech, 10830–1-AP, 1:200), Anxa-3 (Proteintech, 17957–1-AP, 1:200), Fmo-1 (Novus Biologicals, NBP2–16511, 1:100), Fmo-2 (Proteintech, 15725–1-AP, 1:100), Gabrp (Proteintech, 17957–1-AP, 1:100), and Alcam (Proteintech, 21972–1-AP, 1:200). Secondary antibodies conjugated to Alexa Fluor 488 (Invitrogen; 1:100) were incubated overnight at 4°C. Nuclei were counterstained with DAPI, and sections were mounted with Gelvatol. Images were captured using a laser scanning confocal microscope (LSM 710 META; Carl Zeiss).

### Statistical Analysis

The Wilcoxon rank-sum test was used to robustly identify DEGs between the cell clusters. Receiver operating characteristic (ROC) curve analysis, based on the area under the curve (AUC), was used to identify genes with strong discriminatory power between clusters while mitigating sensitivity to data imbalance. Candidate marker genes were selected based on adjusted p-values < 0.05 and AUC > 0.7, indicating both statistical significance and strong discriminative performance. For validation experiments, the Student’s t-test or the Mann–Whitney U test was used to compare gene and protein expression levels between limbal and central corneal epithelial cell groups.

## Results

### High Expression of Sox9-EGFP Positive Cells in Corneal Limbus and Skin Follicles

In the Sox9-EGFP transgenic mice under normal homeostatic conditions, the Sox9-EGFP expression was observed in skin follicles (Fig. 1A). There, discrete EGFP fluorescence was detected primarily around the inner root sheath of hair follicles, especially adjacent to the hair follicle shaft. The most intense EGFP signal was localized to the bulge region of the hair follicles, which corresponds to the known niche of adult skin epithelial stem cells (yellow arrows in Fig. 1A). While in the cornea, clusters of EGFP-positive cells were also observed at the corneal limbus (Fig. 1B). The EGFP-positive cells that formed compact groups were predominantly localized in the inner limbus region. The EGFP-positive cells in the cornea appeared as spherical clusters ranging from 20 to 40 μm in diameter and were discretely distributed with regularly spaced intervals between clusters, which are similar to the distinct structures of the limbal stem cell niche (Fig. 1B). Notably, EGFP-positive cells in both the hair follicles and limbal regions exhibited a small diameter, approximating 5–10 μm, and a high nucleus-to-cytoplasm ratio.

### High Viability of Isolated Corneal Limbal Epithelial Cells Ensures Successful scRNA seq Experiments

Primary corneal epithelial cells were isolated from the limbus of Sox9-EGFP mice under corneal homeostasis (intact corneas). Cellular viability is critical for accurate gene expression profiling. Our analysis revealed consistently high viability rates of limbal epithelial cells across samples, with 98.67 ± 1.00% observed under corneal homeostasis conditions (Table 1). Microscopic examination further confirmed that approximately 1% of EGFP-positive cells were present in the corneal limbal epithelial cell suspension (Fig. 1C).

### The transcriptomic profiles in the corneal limbus.

Two scRNA-seq libraries were constructed to capture the transcriptomic profiles of corneal epithelial cells during epithelial cell homeostasis. Among the two samples, 10,636 and 10,728 cells were detected with mean reads per cell of 39,716 and 40,572, and median genes per cell of 3,618 and 3,487, respectively (Supplementary Table S2). Following quality filtering, 10,152 and 10,199 cells were retained from the corneal homeostasis samples for downstream analysis (Supplementary Table S3). Principal component analysis reduced dimensionality, revealing 13 distinct cell clusters, which were visualized using a t-SNE map (Fig. 1D). Among the cell clusters, the majority of EGFP-positive cells were localized within cluster 12, which exhibited the highest EGFP expression (46.43%) (Fig. 1D). The transcriptomic profile of each cluster is clearly visualized in the heatmap shown in Fig. 2. The distinct genes were identified, as evidenced by the grouping of cells with similar transcriptomic profiles within each cluster. The identification of the top highly expressed DEGs is shown in Supplementary Table S4, which highlights the transcriptomic change that occurs in the corneal limbus epithelium between the cell clusters.

### Cell Population Identification Based on Gene Expression and Cell Cycle Analysis

Gene expression profiles for each cluster were used to determine the cell population type present in each cluster across the two group conditions. Known markers were used to identify LSCs, progenitor cells (PCs), basal epithelial cells (BCs), TA cells, terminally differentiated corneal epithelial cells (TDs), and conjunctival cells (Conj). These markers included Sox9, Cd63, Bmi1, Ctnnb1, Actn1, Gpha2, and Ifitm3 for LSC; Krt14 and Krt15 for PC cells; integrin β1 and β4 for BC cells; Mki67, Birc5, and Cenpa for TA cells; Krt12 and Gjb3 for TD cells; and Muc20 and Krt13 for conjunctival cells. The cell cycle phase for these populations was identified by scoring them using Seurat’s CellCycleScoring function.

Most cell clusters remained in the G0/G1 phase, except for clusters 4, 5, 6, and 7 (Fig. 4B), which were consistent with corneal epithelial cell homeostasis. The majority of cells in cluster 6 were in the S phase, whereas most cells in clusters 5 and 7 were in the G2/M phase (62.1% and 85.3%, respectively). The number of LSCs (clusters 9 and 12) was 1,128 cells, based on high expression of a putative LSC marker and the majority of cells being in the G0/G1 cell cycle stage. Among the LSC clusters, cluster 12 maintained a higher percentage of EGFP-positive cells than cluster 9. Additionally, PC cells (cluster 11) numbered 883 in the corneal limbus epithelium, and TA cell clusters (cluster 5 and 7) expanded significantly to 2,964 in the corneal limbal epithelium. The clusters with the largest numbers of cells were the BC cell cluster (clusters 0, 6, and 10) and the TD cell cluster (clusters 1, 2, and 3), which included 5,354 and 6,969 cells, respectively. The conjunctival cells (cluster 8) were observed in approximately 1310 cells in the corneal limbal samples (Supplementary Table S3). Unfortunately, cluster 4 remained unclassified owing to its mixed gene-transcriptome profile and the presence of multiple cell-cycle stages (Figs. 3A and 3B).

### Different cellular pathways are present in the corneal homeostasis

Cluster 12, which contained most of the LSC-EGFP cells, was compared to all other clusters during the corneal homeostasis. Four top pathways were identified as strongly associated with stem cell maintenance or regenerative functions, including multicellular organismal-level homeostasis, regulation of epithelial cell proliferation, the extrinsic apoptotic signaling pathway, and negative regulation of cell differentiation, among others (Fig. 4A). Additionally, we compared the LSC-EGFP cell cluster with early differentiated cell clusters to identify the most critical genes associated with corneal LSC differentiation. By comparing the LSC-EGFP cell cluster with the PC cell clusters, we identified four additional top pathways that were highly associated with stem cell maintenance, including multicellular organismal-level homeostasis, negative regulation of cell population proliferation, the apoptotic signaling pathway, and negative regulation of cell differentiation, among others (Fig. 4B). By comparing the LSC-EGFP cell cluster with the highly proliferative cell cluster, the TA cluster, we identified four top pathways strongly associated with stem cell maintenance, including multicellular organismal-level homeostasis, regulation of epithelial cell proliferation, the extrinsic apoptotic signaling pathway, and cell-cell adhesion, among others (Fig. 4C). Among the pathways involved in LSC regulation, the multicellular organismal-level homeostasis pathway is a shared pathway that is critical for mediating stem cell fate, regulating tissues, and maintaining homeostasis [35]. The top 25 DEGs for each comparison are listed in Supplementary Table S5 and ranked by average Log_2_ fold change.

### Fmo1, Fmo2, Gabrp, and Alcam Identified as Novel Potential Markers for Limbal Stem Cells

Based on their expression and function, we selected DEGs with a greater potential to contribute to cellular stemness. The selected genes and their related protein function were listed in Supplementary Table S6, which includes Krt8, Krt18, Anxa3, Fmo1, Fmo2, Gabrp, and Alcam. All of these were highly expressed in the Sox9-EGFP mice under corneal epithelial cells’ homeostasis (Fig. 5A). These selected DEGs were further validated by qPCR, Western blotting, and immunofluorescence staining. Gene expression analysis by qPCR confirmed the upregulation of all candidate markers in the corneal limbal epithelial cells when compared with central cornea epithelial cells (Fig. 5C, p < 0.05). Among the qPCR results, Fmo1 exhibited the highest fold change, at 74.46 ± 9.94. Western blot analysis of corneal epithelial cell lysates corroborated the elevated protein expression levels of all these genes in the limbal area compared with central corneal epithelial cells. Among them, Gabrp, Alcam, and Anxa3 showed a significant increase (p < 0.05), with Gabrp showing the highest value at 5.85 ± 1.95 (Fig. 5D and 5E). Immunofluorescence staining further revealed robust expression of Krt8, Krt18, Anxa3, Fmo1, Fmo2, Gabrp, and Alcam in the corneal limbus. Compared with the central cornea epithelial cells, Fmo1, Fmo2, Gabrp, and Alcam were exclusively expressed in the limbus, with only minimal fluorescence detected in the basal epithelial cells of the central cornea (Fig. 6).

## Discussion

In this study, we identified EGFP-expressing cells in the corneal limbus of Sox9-EGFP transgenic mice and established a valuable animal model for investigating corneal limbal stem cell biology. Using scRNA-seq, we profiled approximately 20,364 individual cells isolated from the limbus of Sox9-EGFP mice, thereby delineating a comprehensive transcriptomic atlas and revealing 13 distinct cell clusters during corneal limbal epithelial homeostasis. By leveraging Sox9-EGFP expression, along with established putative stem cell markers and cell-cycling status, we characterized the heterogeneity of limbal stem cells and defined the transcriptome profiles of Sox9-EGFP-positive cells. Using comparative analysis between Sox9-EGFP-positive cells and differentiated corneal epithelial cells, we identified DEGs primarily associated with corneal limbal epithelial homeostasis, thereby enabling the identification of novel LSC markers. These markers were subsequently validated at the transcriptional, protein, and immunofluorescence levels, confirming their exclusive enrichment in the limbal region.

A major challenge in LSCs’ research is the absence of definitive markers for their identification. Some studies have estimated that the adult cornea contains approximately 80–100 LSCs [36], whereas others have reported that LSCs account for approximately 1% to 20% of LSCs based on colony-formation assays [4]. Additionally, the specialized microstructure of the limbus is absent from the mouse cornea, complicating the identification and characterization of LSCs. Despite the lack of these microstructures, we observed EGFP-positive cells localized at the corneoscleral junction in Sox9-EGFP mice. These EGFP-positive cells formed discrete clusters, resembling stem cell clones, each comprising 3–20 cells. These cell clusters were spatially separated throughout the limbus and resembled the confetti-positive clones observed in the Brainbow Confetti mouse limbus, which has been used in previous LSC studies [15]. Such clustered structures may facilitate intercellular support and stem cell maintenance, consistent with the slow-cycling cells reported in our previous study [24].

During scRNA-seq, high-quality sample preparation is critical to ensuring reliable and consistent study results. The sample isolation process can affect cell capture, stress cells, and degrade cytoplasmic transcripts, leading to alterations in cells’ inherent gene expression profiles [37, 38]. A cellular viability greater than 90% is generally considered indicative of high-quality samples for scRNA-seq [39]. In our study, the isolated corneal epithelial cells exhibited excellent viability of 98.67 ± 1.00%, enabling robust downstream transcriptomic analysis. By using the Seurat package in R, we identified 13 transcriptionally distinct clusters. Based on established gene expression, putative stem cell properties, and cell cycle status, these clusters were classified as LSC-EGFP cells, LSC cells, PC cells, TA cells, BC cells, TD cells, and conjunctival cells. Among all epithelial cells, the LSC-EGFP cluster represented only approximately 0.14% of the total cell population. However, when the LSC-EGFP cluster (cluster 12) and the LSC cell cluster (cluster 9) were combined, LSCs accounted for 5.54% of all epithelial cells, which was substantially higher than the previously reported estimate [36, 40]. These findings further demonstrated the heterogeneity of LSCs and further emphasized the importance of identifying a reliable marker for LSCs. PC cells accounted for approximately 4.34% of all cells, which is consistent with their transfer phase from LSC to mature TA cells. In contrast, TD and BC cells accounted for 14.56% and 26.31% of all the cells, respectively. This distribution is consistent with previous reports describing corneal epithelial cell populations during homeostasis.

Through bioinformatic analysis, we observed pronounced heterogeneity of LSCs and the complexity of gene regulatory networks governing corneal epithelial cells from homeostasis. Several putative stem cell regulatory genes, including Krt8, Krt18 [41], Cd63, and Bmi1 [9], which were previously reported to be enriched in LSCs, as well as Sox9, Actn1, and Anxa3, which are common stem cell markers, showed highly expressed patterns in the LSC-EGFP cluster, consistent with previous studies. Validation by qPCR, Western blotting, and immunofluorescence staining further confirmed the high expression of Krt8, Krt18, and Anxa3 in the corneal limbus, consistent with our previous findings in slow-cycling limbal epithelial cells [24]. However, several putative LSC markers exhibited distinct expression dynamics. For example, Gpha2 and Ifitm were more highly expressed in the LSC and PC cell clusters than in the LSC-EGFP cluster during homeostasis (Fig. 3A). These findings suggest that a distinct stem cell subpopulation of LSC-EGFP cells is involved in maintaining corneal epithelial homeostasis and stem cell quiescence. These findings are further supported by the DEGs and pathways analyses, which demonstrated significant enrichment of stem cell-associated pathways when the LSC-EGFP cluster was compared with all other epithelial populations, including PC cells and TA cells (Fig. 4). Among these pathways, the multicellular organismal-level homeostasis pathway, the regulation of cell proliferation pathway, and the cell apoptosis pathway are all highly associated with stem cell maintenance, reinforcing the role of LSC-EGFP cells in sustaining corneal epithelial integrity under physiological conditions. These results reinforce the complexity of gene regulatory mechanisms underlying the renewal of corneal epithelial cells during homeostasis.

By comparing LSC-EGFP cells with PC and TA cells, we identified several genes that might be involved in the transition of limbal stem cells toward differentiated epithelial cells. in addition to Krt8, Krt18 and Anxa3, several other upregulated DEGs, including Fmo1, Fmo2, Gabrp, and Alcam, were identified.

Notably, Gabrp was significantly upregulated in the LSC-EGFP cell cluster compared to that in TA cells. Gabrp, which is involved in inhibitory neurotransmission in the central nervous system, has been reported to activate ERK signaling [42], sustain epidermal growth factor receptor signaling [43], regulate apoptosis [44], induce cell cycle arrest [45], and regulate cell proliferation [46]. Our qPCR (2.39 ± 0.68-fold increasing), Western blot (5.85 ± 1.89-fold increasing), and immunofluorescence staining analyses confirmed its high expression in the corneal limbus. Our work is the first study to demonstrate exclusive Gabrp expression in the corneal limbus, suggesting that Gabrp may contribute to maintaining LSC stemness during corneal epithelial homeostasis.

We also validated the upregulation of Fmo1, Fmo2, and Alcam in corneal limbal epithelial cells. These genes were significantly enriched in biological processes associated with multicellular organismal-level homeostasis pathways. By qPCR, we observed substantial genes expression in the limbus compared to the central cornea, with Fmo1 showing the highest (74.46 ± 9.94-fold), followed by Fmo2 (14.8 ± 0.31-fold) and Alcam (1.87 ± 0.14-fold). Although Western blotting detected strong expression only for Alcam, immunofluorescence staining confirmed the markedly elevated expression of all three genes in the corneal limbus. Fmo1 and Fmo2 encode flavin-containing monooxygenases, which are variably expressed during development. They are involved in oxygenation reactions, oxidative stress resistance, and the promotion of homeostasis through metabolic remodeling [47–49]. Notably, Fmo2 was recently reported as a putative active LSCs marker, exhibiting approximately 74-fold higher expression in enriched human LSCs than differentiated corneal epithelial cells [50]. Our findings suggested that Fmo1 and Fmo2 may help maintain LSCs’ stemness by enhancing cellular adaptation to oxidative stress. In addition, Alcam (activated leukocyte cell adhesion molecule), is a well-established regulator of hematopoietic stem cell self-renewal [51] and is widely recognized as a marker of human mesenchymal stem cells [52]. Here, we first demonstrated high Alcam expression in the corneal limbus, suggesting that it may be a novel candidate marker for LSCs.

## Conclusion

This study employed scRNA-seq using Sox9-EGFP as a reference marker, establishing an effective animal model for investigating limbal stem cells. The transcriptomic profiling of Sox9-EGFP-positive cells revealed several novel candidate LSCs markers, including Gabrp, Fmo1, Fmo2, and Alcam, which were subsequently validated at both the transcriptional and protein levels. These findings provide new insights into the molecular characteristics of LSCs and establish a comprehensive transcriptomic atlas that highlights their cellular heterogeneity during corneal epithelial homeostasis.

## Supplementary Material

Supplementary Files

This is a list of supplementary files associated with this preprint. Click to download.

- Supplementary.pdf

## Figures and Tables

**Figure 1:**
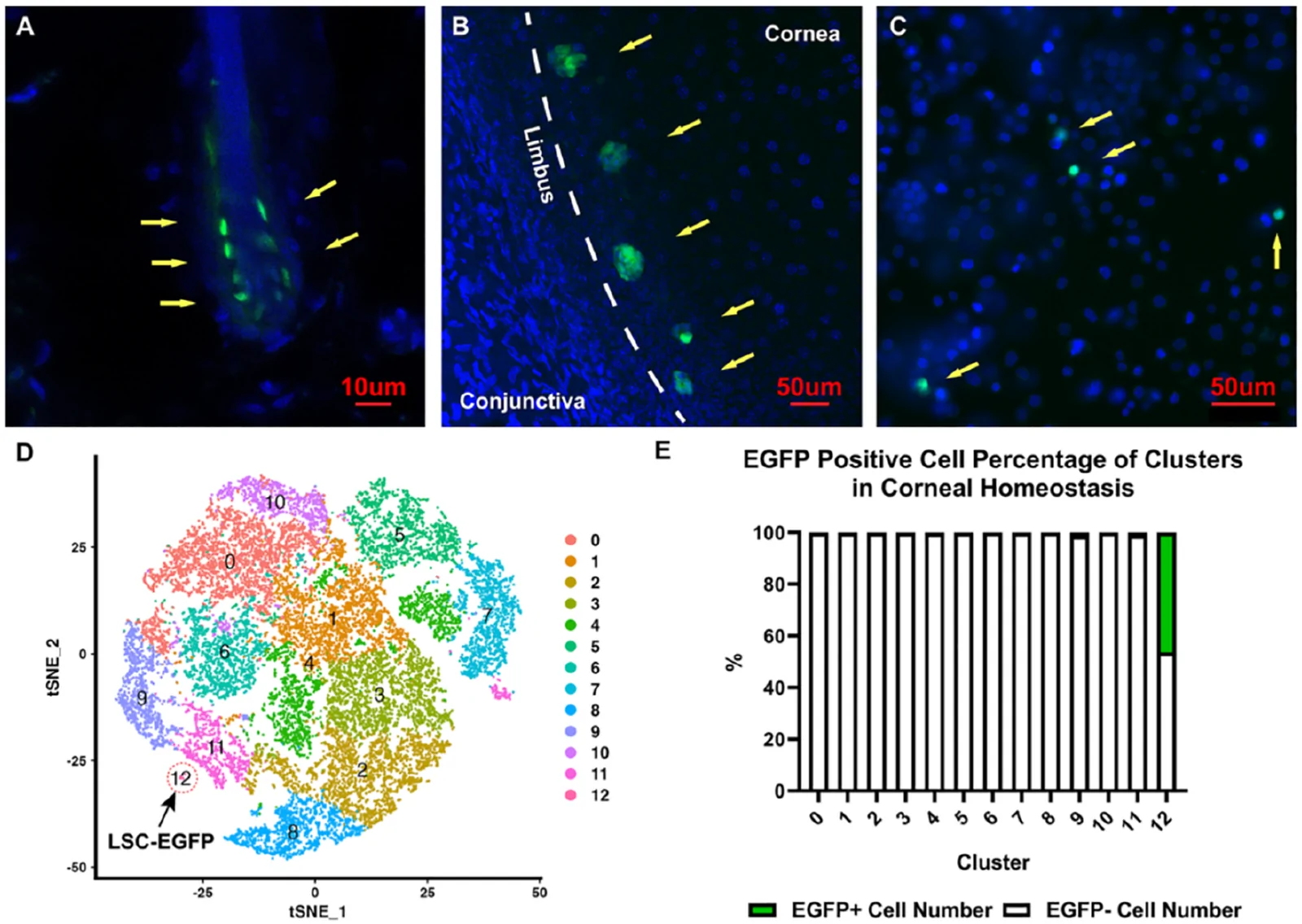
EGFP-positive cells were isolated from Sox9-EGFP mice and identified by scRNA-seq analysis. The Sox9-EGFP-positive cells can be visualized by immunofluorescence in the skin and cornea of Sox9-EGFP mice, allowing their isolation and purification for single-cell RNA sequencing. (A) EGFP-positive cells were detected in hair follicle sections of Sox9-EGFP mice, with high expression in the hair follicle bulge region. (B) EGFP-positive cells were visualized in whole-mount images of the corneal limbus during homeostasis (intact corneas). (C) EGFP-positive cells were observed after isolation of corneal limbal epithelial cells from corneas. Among these cells, approximately 1% were EGFP-positive, and cell aggregates accounted for approximately 17.85 ± 10.11% of the total cell population. (D) t-SNE map displays the single-cell transcriptomic profiles of corneal limbal epithelial cells collected from Sox9-EGFP mice under corneal homeostasis (21,066 cells with 98.67 ± 1.00% viability). Each sample (n=2) comprised cells pooled from five mouse corneas. (E) Within these datasets, EGFP gene expression was enriched in cluster 12, with 46.43% of the cells expressing EGFP under corneal homeostasis. Yellow arrows point to the EGFP-positive cells.

**Figure 2:**
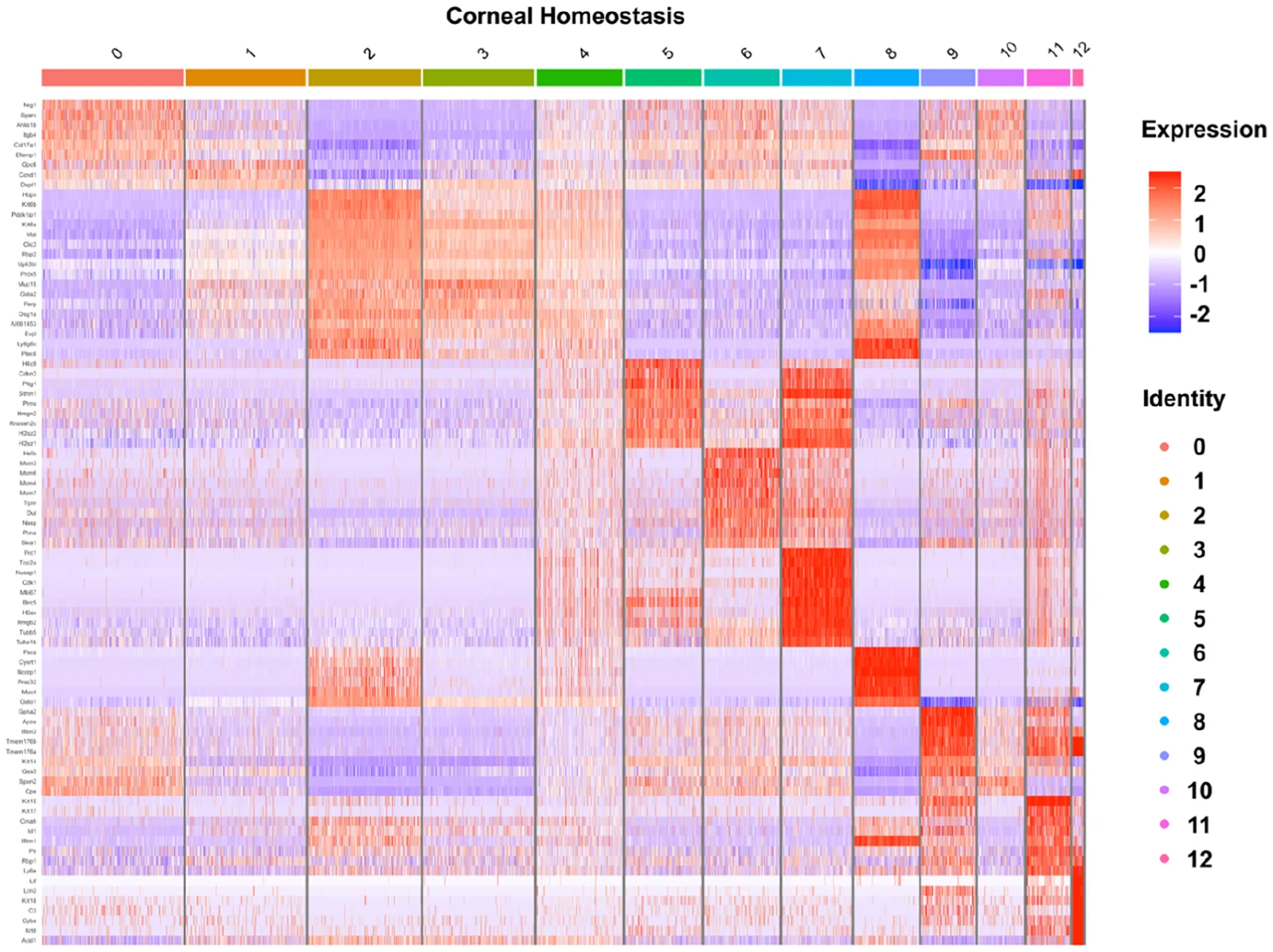
The transcriptomic profiles in the corneal limbus. The heatmap of the top 10 highly expressed DEGs was categorized among 13 cell clusters, demonstrating distinct cell population patterns from the limbus of the cornea (A). Changes in the gene expression profile are evidenced by the color assigned during the analysis.

**Figure 3:**
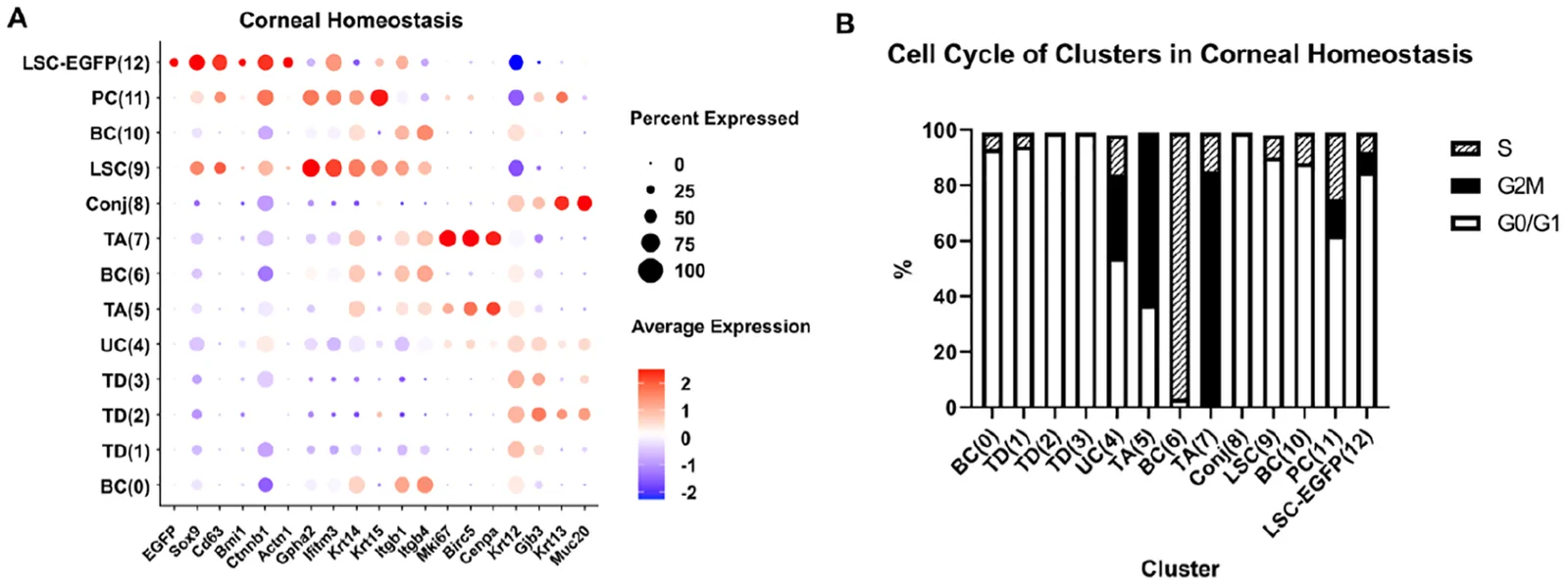
Identification of cell populations present in each cluster based on gene expression similarity. We used the gene expression profile to identify the cell type present in each cluster derived from the corneal limbal epithelium during homeostasis (A). Dot plots show the expression of classical marker genes for each cluster with distinct cell populations identified, including limbal stem cells with a high proportion of EGFP-positive cells (LSC-EGFP), limbal stem cells (LSC), progenitor cells (PC), basal cells (BC), transit-amplifying cells (TA), terminally differentiated cells (TD), and conjunctival cells (Conj). Among these, cluster 12 exhibited strong EGFP expression along with putative stem cell genes. (B) During homeostasis, clusters 5 and 7 contained 62.12% and 85.31% of cells in the G2/M phase of the cell cycle. The cluster axis indicates the assigned cell type, with original cluster numbers shown in parentheses.

**Figure 4:**
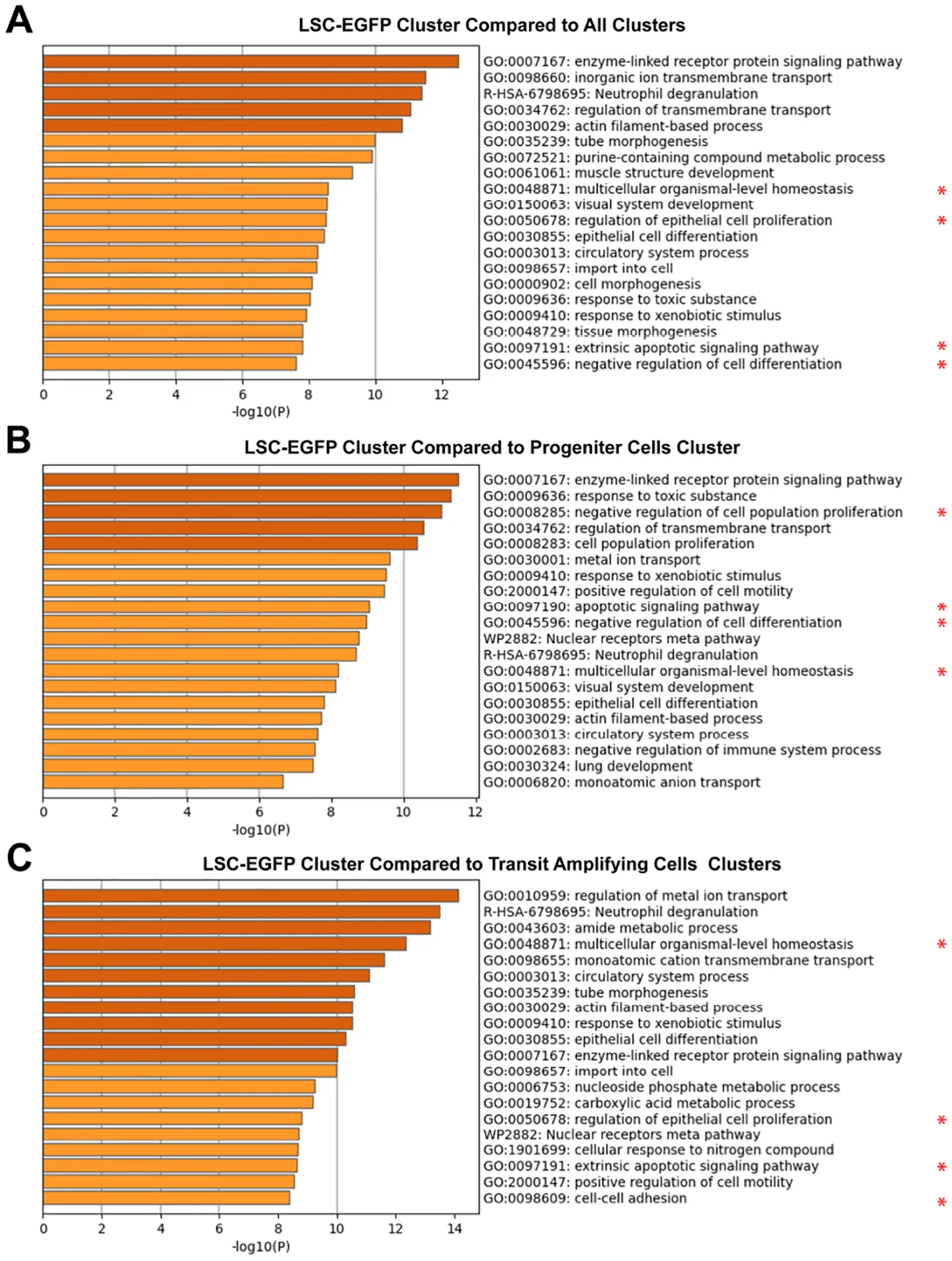
Cellular pathways strongly associated with stemness are present in corneal homeostasis. The DEGs of LSC-EGFP cells from cluster 12 were compared to those of all other clusters (A), PC clusters (B), and TA clusters (C). This analysis identifies the pathways that are present in the LSC-EGFP cell cluster. Pathways marked with an asterisk (*) are highly associated with stem cell functions.

**Figure 5:**
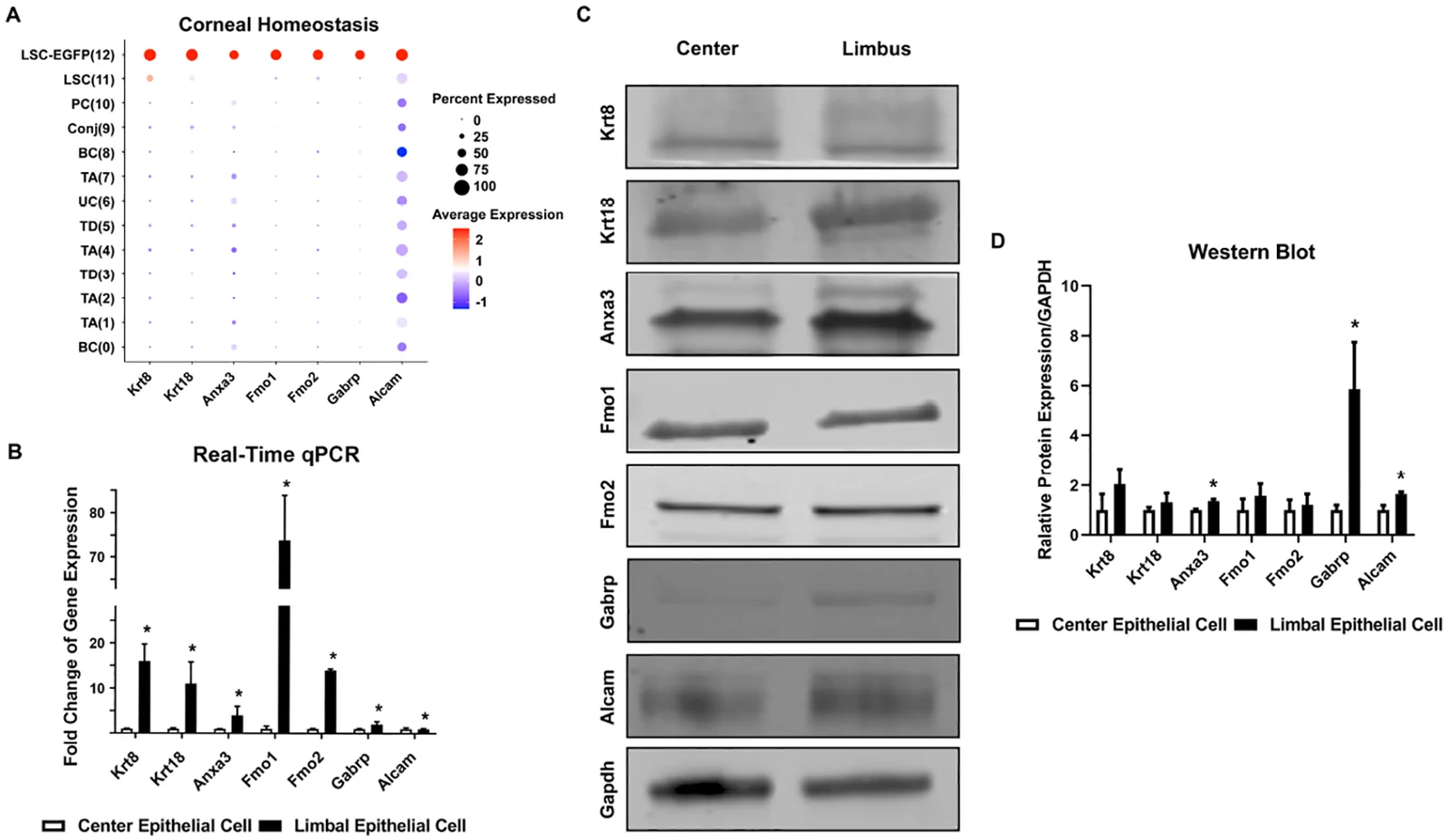
Identification and validation of novel potential marker genes for limbal epithelial stem cells. scRNA-seq analysis identified seven genes-Krt8, Krt18, Anxa3, Fmo1, Fmo2, Gabrp, and Alcam-as highly expressed in LSC-EGFP clusters under corneal epithelial homeostasis (A). Validation using quantitative PCR on CD1 mice showed significantly higher expression of these genes in the limbal corneal epithelium compared with the central cornea epithelium (B). At the protein level, Gabrp, Alcam, and Anxa3 were significantly enriched in the limbal corneal epithelium compared with the central cornea epithelium (C and D). Data are presented as mean ± standard deviation (SD) (n = 3, p < 0.05). (The original Western blots are presented in the Supplementary Figure S1.)

**Figure 6:**
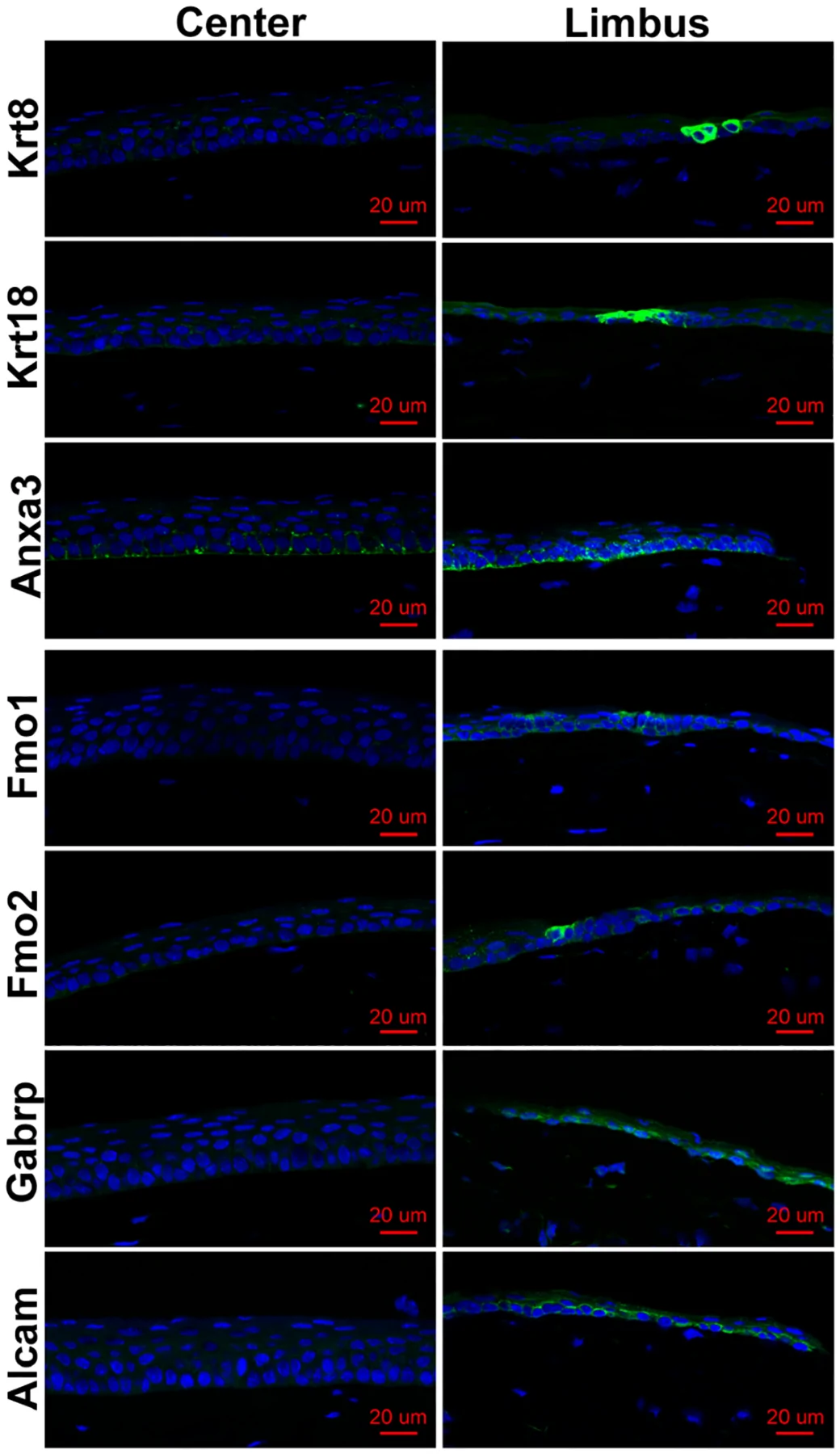
Validation of novel potential marker genes for limbal epithelial stem cells by immunofluorescence staining. Corneal cryosections from CD1 mice were used to validate the genes identified as highly expressed in the limbus using scRNA-seq. We found that Krt8, Krt18, Anxa3, Fmo1, Fmo2, Gabrp, and Alcam were highly expressed in the limbus. Among these genes, Fmo1, Fmo2, Gabrp, and Alcam were identified as exclusively expressed in the limbus.

**Table 1:** Summary viability and aggregation of isolated corneal epithelial cells presented in the samples used for scRNA-seq.

Sample	Viability (%)	Aggregation (%)
Sample 1	98.08	10.70
Sample 2	99.50	25.00
Mean	98.67	17.85
Standard Deviation	1.00	10.11

## Data Availability

The scRNA-seq fastq raw data were deposited in the Sequence Read Archive (SRA) repository with accession number PRJNA1454550. The data is currently under controlled access. Reviewer access has been provided via the following link: https://dataview.ncbi.nlm.nih.gov/object/PRJNA1454550?reviewer=j2iah9h7ersi64jp5t3j1lnrq2.
